# Schema Therapy for Children and Adolescents: A Systematic Review of Individual, Group, and Parent-Based Interventions

**DOI:** 10.1007/s10567-026-00568-4

**Published:** 2026-04-16

**Authors:** Melike Uysal, Ipek Ege Gurel Ficicioglu, Muge Mutlu, Tuba Mutluer

**Affiliations:** 1 Department of Child and Adolescent Psychiatry, Kirikkale High Specialization Hospital , Kirikkale, Turkey; 2 Department of Child and Adolescent Psychiatry, Bakirkoy Prof. Dr. Mazhar Osman Training and Research Hospital for Neurological Diseases and Mental Health, Istanbul, Turkey; 3 Department of Child and Adolescent Psychiatry, Silivri State Hospital, Istanbul, Turkey; 4 Department of Child and Adolescent Psychiatry, Akdeniz University School of Medicine, Antalya, Turkey; 5 Research Center for Translational Medicine (KUTTAM) and Neuroscience Research Program, Koç University, Istanbul, Turkey

**Keywords:** ST-CA, Schema therapy, Child, Adolescent, Parent, Psychotherapy

## Abstract

Schema therapy (ST) is an integrative psychotherapy targeting early maladaptive schemas formed in childhood. Recently, ST has been increasingly used to treat emotional, behavioral, and interpersonal difficulties in youth. This review synthesized evidence on ST interventions for individuals aged 5–18 across individual, group, and parent-focused formats. Following PRISMA 2020, a systematic search of PubMed, Cochrane Library, Scopus, Web of Science, and Ovid MEDLINE (2000–2025) identified studies using schema therapy or schema-informed interventions with quantitative outcomes from quasi-experimental or pre–post designs. Twenty-one studies met inclusion criteria: five individual, nine group, and eight parent- or family-based interventions (one overlapping). This review is registered with PROSPERO (CRD420251064229, May 30, 2025). Across modalities, schema therapy was associated with improvements in emotional regulation, depressive and anxiety symptoms, behavioral difficulties, and interpersonal functioning across studies. Group and individual formats reduced emotional and behavioral problems, enhanced coping, and improved social functioning. Even brief programs with few sessions were associated with meaningful improvements, with several studies showing maintained gains at short-term follow-up. Parent-focused interventions were associated with improvements in children’s emotional and behavioral outcomes while enhancing parental insight, self-efficacy, and family functioning. However, notable variability in treatment duration, outcome measures, and intervention structures remained across studies. Schema therapy shows promising efficacy for emotional, behavioral, and relational outcomes in youth, with parent involvement enhancing effects. Yet, heterogeneity in protocols and implementation underscores the need for standardization. Future studies should use standardized, randomized, and longitudinal designs to strengthen evidence for developmentally tailored schema therapy.

## Introduction

Schema therapy (ST) is an integrative psychotherapy developed by Young in the early 1990s that focuses on identifying and changing maladaptive schemas and coping patterns. In light of mounting evidence that the foundations of schemas are established in the context of early caregiving and parent and also peer relationships, there has been an increase in interest in modifying ST for developmental populations during recent years. Because of its developmental plausibility and its explicit emphasis on needs, emotions, and relational experiences, ST is positioned as a potential transdiagnostic intervention for young people who frequently exhibit a range of symptoms (Young et al., 2006). Over the past years, growing evidence has supported the effectiveness of ST in adults, particularly in the treatment of personality disorders. Randomized controlled trials (RCTs) and meta-analytic findings have demonstrated the efficacy of ST for personality disorders, highlighting its impact on symptom reduction and functional outcomes (Bamelis et al., 2014; Masley et al., 2012). Schema therapy has increasingly been adapted for younger populations, with emerging studies exploring its application in children and adolescents through tailored interventions that include experiential techniques, imagery-based work, and family involvement (Loose & Pietrowsky, 2016; Roelofs et al., 2016).ST has been proposed as an intervention that may contribute to improvements in emotional, behavioral, and relational functioning by targeting schema-related beliefs and mode patterns. It also places a strong emphasis on the therapeutic relationship while incorporating aspects of other therapeutic approaches, such as imagery rescripting (IR). In ST, IR is used to help individuals reinterpret distressing memories in a way that strengthens adaptive self-regulation (“healthy adult” functioning), provides emotional support to unmet childhood needs (“vulnerable child” states), and reduces rigid or avoidant coping responses that maintain psychological difficulties (Young et al., 2006). Through guided imagery, individuals are encouraged to re-experience past interpersonal or traumatic events and reconstruct them from a new, healing perspective.

Early maladaptive schemas (EMSs) are pervasive cognitive, emotional, and interpersonal patterns that begin to develop in childhood and influence later functioning throughout life (Young et al., 2006). According to schema perspective, psychological difficulties emerge when essential emotional needs in early life, including secure attachment, are not adequately met. This deprivation leads to the development of early maladaptive schemas, whose activation elicits schema modes associated with dysfunctional emotions and behaviors. Although EMSs may appear functional in ensuring a child’s psychological survival in less-than-optimal contexts, over time they may increase vulnerability to psychopathology (Farrell et al., 2014).

Schema therapy for children and adolescents (ST-CA) represents a developmentally sensitive adaptation of the original adult model. Rather than directly translating adult protocols, ST-CA modifies conceptual language and techniques to match children’s and adolescents’ cognitive and emotional capacities, relying more heavily on concrete and experiential methods (e.g., imagery, simplified mode work, activity-based techniques). Given that schemas are still consolidating during childhood and adolescence, interventions place greater emphasis on emotion regulation and relational needs (Graaf & Loose 2013; Young et al., 2006). ST-CA may be a pivotal intervention as it’s possible that EMSs may be less consolidated in children than in adults, which suggests that focused techniques can produce significant change. Moreover, since the development of children’s schemas is closely shaped by caregiver schemas and parent–child interaction patterns, interventions that include parents and target parenting styles may have broader systemic benefits (Louis et al., 2020).

Furthermore, youth mental health services increasingly require developmentally attuned, mechanism-based, and scalable interventions that can be integrated into school, outpatient, and community settings. Schema therapy—given its transdiagnostic nature, adaptability across developmental stages, and inclusion of caregiver-focused components—holds strong potential to bridge the gap between evidence-based mechanisms and real-world service delivery (Graaf & Loose ).

A recent systematic review by Joshua et al. (2025) synthesized applications of schema therapy in young people and highlighted the emerging yet heterogeneous nature of the evidence base (Joshua et al., 2025). The present systematic review aims to extend the existing literature by focusing specifically on children and adolescents and by systematically assessing the efficacy of schema therapy across individual, group, and parent-/family-focused formats. Quantifying and systematically synthesizing clinical outcomes related to ST across formats, settings, and youth presenting problems and analyzing evidence for change in schema-relevant processes, such as early maladaptive schemas, modes, emotion regulation, and relational functioning, are other highlights of this study.

The aim of this systematic review was to systematically identify and synthesize empirical studies examining schema therapy interventions in children and adolescents. Specifically, the review evaluates the reported clinical outcomes of schema therapy across individual, group, and parent-focused intervention formats. By integrating findings across these modalities, the review seeks to provide a developmentally informed synthesis of the current evidence and to examine potential mechanisms of change associated with schema therapy in youth populations.

## Methods

### Search Strategy

This systematic review was conducted in accordance with the Preferred Reporting Items for Systematic Reviews and Meta-Analyses (PRISMA) 2020 guidelines, as outlined by Page et al. (Page et al., 2021). Following PRISMA 2020 guidelines, a systematic search was conducted in PubMed, Cochrane Library, Scopus, Web of Science, and Ovid MEDLINE for studies published between 2000 and 2025. Eligible studies implemented schema therapy or schema-informed interventions and reported quantitative outcomes using randomized, quasi-experimental, or pre–post designs. Twenty-one studies met inclusion criteria: five individual interventions, nine group interventions, and eight parent-based or family-involved interventions (one overlapping). This systematic review is registered with PROSPERO (CRD420251064229, May 30th, 2025). Two independent reviewers screened the titles and abstracts of all identified records, followed by full-text assessment of potentially eligible articles. Any disagreements were resolved through discussion and consensus, with a third reviewer consulted when necessary. The level of agreement between reviewers during the screening stages was high. The full search strategies are provided in Appendix 1.

### Study Design and Reporting Framework

This systematic review was conducted in accordance with the PRISMA 2020 guidelines (Page et al., 2021). Its primary aim was to evaluate the effectiveness of ST interventions for children and adolescents aged 5–18 years across individual, group, and parent-focused formats.

ST is an integrative psychotherapy approach designed to identify, modify, and replace EMSs—deeply rooted cognitive, emotional, and behavioral patterns that originate in childhood and influence later psychological functioning. In recent years, ST has been increasingly applied to address emotional, behavioral, and interpersonal difficulties in youth, and the present review synthesizes the available empirical evidence regarding its effectiveness across different intervention formats. In this systematic review, studies were included if the intervention was explicitly described as schema therapy and was grounded in Young’s schema therapy framework. Interventions were considered eligible when they targeted early maladaptive schemas and/or schema modes and incorporated characteristic schema therapy techniques, such as limited reparenting, experiential techniques (e.g., imagery rescripting or chair work), or cognitive-behavioral schema-focused strategies.

The search was limited to studies published from 2000 onward, as schema therapy began to be more systematically described and applied in clinical research during this period (Young et al., 2006). However, to ensure that potentially relevant earlier studies were not overlooked, records published prior to 2000 were also screened during the initial search process; no eligible studies from this period were identified.

### Eligibility Criteria

Inclusion criteria encompassed peer-reviewed articles evaluating the effectiveness of schema therapy in children and adolescents. Studies were included if they met the following conditions:

(i) Original research articles involving participants aged 5 to 18 years;
(ii) Studies applying schema therapy as a structured psychotherapeutic intervention, either individually or in group formats;
(iii) Quantitative study designs, including RCTs, quasi-experimental studies, or pre-post evaluations;
(iv) A minimum sample size of four participants to allow inclusion of small clinical case series and early-phase intervention studies, given the emerging nature of schema therapy research in younger populations (However, findings from studies with very small samples were interpreted cautiously and considered exploratory rather than confirmatory in nature.);
(v) Articles published between 2000 and 2025 in peer-reviewed journals;
(vi) Publications written in English.

### Exclusion Criteria

Studies were excluded if they met any of the following conditions:

(i) Articles not presenting original research, such as reviews, theoretical papers, or meta-analyses;
(ii) Materials such as conference abstracts, editorials, commentaries, opinion pieces, case reports, preprints, and study protocols;
(iii) Studies involving only adult populations or mixed-age samples without separately reported data for participants aged 5–18;
(iv) Articles not focusing on schema therapy as the primary intervention or lacking adequate methodological details about the therapeutic procedures;
(v) Studies not reporting quantitative clinical outcomes or lacking pre- and post-intervention data;
(vi) Studies with fewer than four participants in total;
(vii) Publications written in languages other than English.

These inclusion and exclusion criteria were designed to ensure the selection of methodologically sound and clinically relevant studies that directly address the research question of this systematic review.

### Data Extraction and Analysis

Data from all eligible studies were systematically extracted and organized to enable structured comparisons across key variables. Extracted information included authorship and year of publication, total sample size, participant demographics (age range and gender distribution), and clinical characteristics of the target population (e.g., adolescents with behavioral problems, children with emotional dysregulation). Details of the intervention were also recorded, including format (individual vs. group), duration, treatment setting, and the presence of parental involvement.

Outcome measures (e.g., standardized rating scales, clinician-administered assessments) and main quantitative findings were extracted to enable comparative analysis. Data extraction was performed independently by two reviewers using a standardized form covering study design, sample characteristics, intervention features, outcome domains, and key results. Before full extraction, a subset of studies was jointly reviewed to ensure consistency in coding and interpretation. Discrepancies were resolved through discussion and consensus.

This systematic and consensus-based approach facilitated a comparative and thematic synthesis of the findings, identifying consistent trends, methodological heterogeneity, and preliminary evidence regarding the clinical applicability and effectiveness of schema therapy in child and adolescent populations.

## Quality Assessment

The methodological quality of the included studies was independently assessed by three authors using the Joanna Briggs Institute (JBI) Critical Appraisal Tools, selected according to the study design of Munn et al. (2014). As no RCTs met the revised inclusion criteria, quality assessment was conducted using the JBI Checklist for Quasi-Experimental Studies and the JBI Checklist for Case Series, as appropriate. These tools evaluate 9 methodological domains depending on study type, including clarity of objectives, appropriateness of sampling, measurement validity, and adequacy of statistical analysis.

Study quality was categorized according to the percentage of criteria met as follows: Low (≤ 50%), Moderate (51–69%), and High (≥ 70%). These thresholds were defined by the authors in line with conventions used in previously published systematic reviews (Azmiardi et al., 2022; Nasuuna et al., 2024).

## Results

**Fig. 1:**
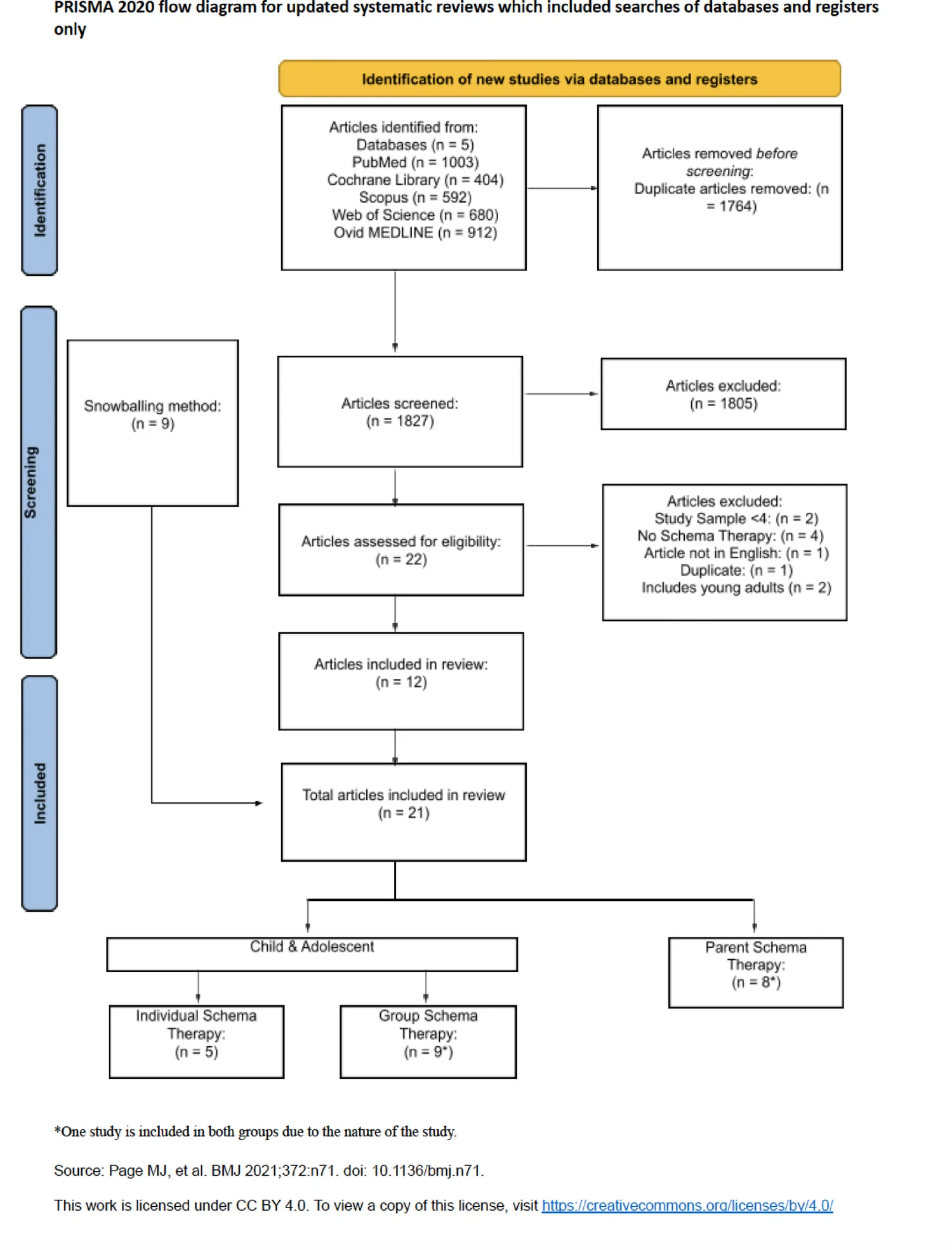
PRISMA flowchart for study selection process

In total, 21 studies met the inclusion criteria. A flowchart of the study selection process following the PRISMA 2020 framework is presented in Fig. 1. The included studies encompassed a broad range of schema therapy applications for children and adolescents reflecting growing interest in adapting this model for developmental contexts.

Although the studies were initially grouped into three main categories (individual schema therapy interventions, group schema therapy programs, and parent-focused or family-involved approaches), several overarching patterns emerged across modalities. Across intervention types, studies examining schema therapy generally reported improvements in emotional regulation, behavioral adjustment, and interpersonal functioning. Interventions incorporating experiential and imagery-based components were often associated with improvements in emotional insight and adaptive coping. Parent-involved models strengthened therapeutic outcomes by addressing maladaptive parenting schemas and improving family dynamics.

Despite differences in treatment format and duration, common mechanisms such as emotional awareness, reparenting, and corrective relational experiences appeared central to therapeutic change, suggesting that the quality and developmental sensitivity of the therapeutic process may be more influential than the specific intervention format itself.

### Individual Schema Therapy

A total of five studies examined individual ST-CA interventions, including a combined sample of 166 participants aged between 12 and 18 years. The average number of sessions administered across the included studies was 14.6. Across these studies, individual schema therapy interventions were associated with improvements in anxiety, depressive symptoms, behavioral problems, and early maladaptive schemas, indicating therapeutic benefits across emotional and behavioral domains (Alboushoke et al., 2024; Karimipour et al., 2022; Van Wijk-Herbrink et al., 2017). The reported effect sizes were generally within the medium-to-high range, with moderate-to-large effects reported across the included studies (Karimipour et al., 2022; Schmied et al., 2024).

Several studies (3 of the 5 included trials) provided follow-up data, with most reporting maintained therapeutic gains over 2–3 months.(Alboushoke et al., 2024; Kheiripour et al., 2023; Schmied et al., 2024). Additionally, the limited number of sessions applied across studies is noteworthy, as several interventions achieved significant outcomes despite their brevity. For instance, Schmied et al. (2024) reported significant pre–post improvements in emotional regulation, self-efficacy, depressive and anxiety symptoms, as well as reductions in non-suicidal self-injury following a brief two-session IR intervention. Importantly, these gains were maintained at three-month follow-up. However, given the absence of a comparison group and the small sample size, these findings should be interpreted as preliminary and not as definitive evidence of efficacy. Collectively, these findings indicate that even short-term, developmentally tailored schema therapy applications appear to be associated with substantial emotional and behavioral improvements among children and adolescents (Table 1).

**Table 1:** Individual schema therapy interventions in children and adolescents

Reference	Aim	Study design	Sample characteristics	Schema therapy format	Follow-up	Assessment tools	Results
Alboushoke et al., (2024)	EST efficacy on anxiety sensitivity & body-checking in girls with SAD	Quasi- ET	*n* = 25; Female ratio: 100%; Age range: 15–18; M = 16.46 ± 2.59	EST 10 weekly sessions (90 min each)	2-month follow-up	SPIN, ASI, BCQ	Reductions in anxiety sensitivity and body-checking maintained at follow-up (*p*<.001)
Kheiripour et al., (2023)	Emotion regulation model (ERT + EST+ETT) efficacy in internet addiction & brain/behavioral systems	Quasi- ECT	*n* = 100; TG = 48, CG = 52; Female ratio = 100%; Age range: 15–18, M = 15.18 ± 1.31	EST 7 sessions (90 min each)	3-month follow-up	IAQ, r-RSQ, GHQ	Reduction in internet addiction after emotion regulation training (*p*=.001); large effect sizes (η²=0.73–0.87)
Schmied et al., (2024)	Two-session IR efficacy & feasibility for NSSI & related symptoms in adolescents	Multiple Case Series	*n* = 7; Female Ratio: 86%; Age range: 14–17; M = 15.6;	IR 2 sessions (90 min each)	3-month follow-up	ERQ, WIRKALL_r, self-report diaries, BDI-II, STAI-T, BIKEB	Improvements in adaptive emotion regulation, self-efficacy, depression and anxiety (*p*≤.01, η²=1.1–2.8) up to 3 months post-intervention; NSSI frequency decreased (*p*≤.05, η²=0.75–0.93)
Karimipour et al., (2022)	ST-CA effectiveness on internalizing problems in adolescents	Quasi-ET	*n* = 30; TG = 15, CG = 15; Female Ratio: 63%; Age Range:12–14; M(TG) = 13.70 ± 1.30; M(CG) 13.33 ± 0.81	ST-CA 14 weekly sessions (120 min each)	-	CBCL	Internalizing problems (total) reduced (*p*<.001, η²=0.74); reductions in anxiety–depression (*p*<.001, η²=0.77), withdrawal–depression (*p*<.001, η²=0.47), somatic complaints (*p*<.001, η²=0.64)
Van Wijk-Herbrink et al., (2017)	Residential ST effectiveness for disruptive behaviors & PD traits in adolescents	Multiple Case Series	*n* = 4; Female Ratio: 50%; Age range: 15–17	ST 9–12 months; minimum 40 sessions; 1–2 sessions per week	-	MOS, YSQ-A, YSR, CBCL	Behavior problems, maladaptive schemas, and schema modes improved; significance assessed via RCI (no group-level p or effect size reported)

ASI: Anxiety Sensitivity Index; BCQ: The Body Checking Questionnaire; BDI-II: The Beck Depression Inventory; BIKEB: The Bielefeld Client Experience Questionnaire; CBCL: Child Behavior Checklist; CG: control group; ECT; Experimental Trial; EMS: early maladaptive schemas; ERQ: Emotion Regulation Questionnaire; ERT: Emotion Regulation Therapy; EST; Emotional Schema Therapy; ETT: Emotional Transformation therapy; GHQ: Goldberg and Hiller’s General Health Questionnaire; IAQ: Young’s Internet Addiction Questionnaire; IR: Imaginary Rescripting; MOS: Mode Observer Scale; NG: Not Given; NSSI: Non-Suisidal Self-Injury; PD: Personality Disorder; RCI: Reliable Change Index; r-RSQ: Jackson’s Revised Reinforcement Sensitivity Questionnaire; SAD: Social Anxiety Disorder; SAQ: Pike and Wiseman Social Adjustment Questionnaire; SASA: Social Anxiety Scale for Adolescents; SPIN: The Social Phobia Inventory; STAI-T: The State–Trait Anxiety Inventory-Trait Version; ST-CA: Schema Therapy for Child and Adolescent; TG: Treatment Group; YSQ-SF3: Young Schema Questionnaire- Short Form 3; WIRKALL_r: General Self-Efficacy Expectancy Scale; YSQ-A: Young Schema Questionnaire for Adolescents; YSR: Youth Self Report; η²: Effect Size

### Group Schema Therapy

A total of nine studies investigated group-based ST-CA interventions. One of these studies included both child-focused group schema therapy and parent-focused intervention components and therefore was incorporated into both the group-based and parent-based approaches categories of this review (Laious et al., 2024). The total sample across these studies comprised 434 participants, with ages ranging from 9 to 19 years. The average number of sessions administered across the included studies was 15.3.

Group-format ST-CA was associated with improvements in social adaptation, academic stress, and behavioral regulation, with positive outcomes reported in most studies (7 out of 9), while a minority yielded mixed findings or no superiority over comparators (Ataei et al., 2024; GhodsiKhorasani et al., 2024; Koçak & Çelik, 2023; Rasouli Saravi et al., 2020). Additionally, significant reductions in early maladaptive schemas were reported, indicating positive cognitive and emotional restructuring processes (Laious et al., 2024; Roelofs et al., 2016). In Rijo et al. (2020), group schema therapy for adolescents with personality disorder features did not yield significant reductions in total EMS scores; however, marked improvements were observed in schema-related emotions, suggesting that emotional restructuring and awareness may occur earlier than cognitive schema change during the therapeutic process. Reported effect sizes in these studies generally ranged from medium to high, supporting the clinical significance of these findings (Rijo et al., 2020).

Although the majority of studies (89.9%) employed quasi-experimental designs, the results collectively indicate that group schema therapy appears to be associated with clinical improvement and enhanced social functioning among children and adolescents (Table 2).

**Table 2:** Group schema therapy interventions in child and adolescents

Reference	Aim	Study design	Sample characteristics	Schema therapy format	Follow-up	Assessment tools	Results
Ataei et al., (2024)	Group ST vs. CBM effectiveness on test anxiety in male adolescents	Quasi- ET	*n* = 45; ST = 15, CBM = 15, CG = 15; Female ratio: 0%; Age: NG	GST 8 weekly sessions (90 min each)	-	ATI, PDT, ABEP	Differences between ST, CBM, and control groups in test anxiety (*p*=.001)
Hasani et al., (2020)	Parental training & ST efficacy on aggression & ODD in adolescents	Quasi- ET	n: 45; ST = 15, PBMT = 15, CG = 15; Age range: 13–16	GST 8 weekly sessions (90 min each)	-	BPAQ, CDQ	PBMT more effective than GST in reducing aggression and oppositional defiant behavior (*p*<.01)
L. Koçak & E. Çelik, (2023)	Group ST counselling effectiveness on academic expectation stress in adolescents	Quasi- ET	n: 20; TG = 10, CG = 10; Female Ratio:0%; Age Range: 14–16; M = 15.2	GCST 12 sessions (90 min each)	follow-up, duration NG	AESI	Internet addiction scores decreased in the experimental group after emotion regulation training (*p*<.01)
Laious et al., (2024)	ST-based parent–child group program efficacy for preventing child psychopathology	Quasi- ET	*n* = 90; Female ratio: 47.8%; Age Range: 9–13; Mean Age: 11.2 ± 1.0	GST children: 16 weekly sessions; parents 10 biweekly sessions (90 min each)	-	SDQ-PV, SDQ, YSQ-SF3, SIC	Child EMS mostly decreased (*p*<.01, *r*=.19–0.27); many parent EMS decreased (*p*<.05, *r*=.16–0.25); reductions in child emotional symptoms (*p*=.001, *r*=.21), peer problems (*p*=.006, *r*=.22), total difficulties (*p*=.005, *r*=.22); increase in prosocial behavior (*p*=.02, *r*=.19)
Rijo et al., (2020)	Group ST efficacy for older adolescents with PD features	Quasi- ET	n: 123; Female Ratio: 0%; TG = 63, CG = 60; Age range: 14–19	GST 25 biweekly sessions	-	SAIAS‑AB	No difference in EMS total scores (*p*=.743; all subscales *p*>.05); SRE decreased (*p*<.01, most *p*<.001, η²*p*=.084–0.27, medium–large effects)
Rasouli Saravi et al., (2020)	GST effectiveness on eating attitude & self-regulation in overweight female adolescents	Quasi- ET	n: 30; TG = 15, CG = 15; Female Ratio: 100%; Age Range:15–17	GST 13 weekly sessions (90 min each)	3 months	YSQ-SF, OS, EAQ, SRS	Self-regulation and eating attitudes improved in GST group (*p*<.001), maintained at follow-up
Roelofs et al., (2016)	GST applicability in adolescents with PD or PD traits	Multiple Case Study	n: 4; Female Ratio: 75%; Age Range:16–18; M: 17.25 ± 0.96	GST 6–18 months; weekly or every 2 weeks	-	Kidscreen-10, SDQ, SMI (adults), TSMI, YSQ-A, SCI, STCRS	Maladaptive modes decreased; healthy modes increased across therapy (reported p values < 0.05)
GhodsiKhorasani et al., (2024)	ST effectiveness on anxiety & social adaptation in delinquent adolescents	Quasi- ET	*n* = 32; TG = 16; CG = 16; Female Ratio: 0% Age Range:14–18; M(TG) = 15.63 ± 1.50; M(CG) = 16.00 ± 1.54	GST 12 sessions	Follow-up reported, duration/ details not specified	SASA, SAQ	Social anxiety and social adaptation improved in treatment group (*p*<.05)
Salari et al., (2023)	GST vs. SST effectiveness on social competence & aggression in male adolescents with ODD	Quasi- ET	n: 45; GST = 15, SST = 15, CG = 15; Female Ratio :0%; Age Range: NG	GST 20 sessions (60 min each)	-	SCQ, AQ	GST and SST equally improved social competence and reduced aggression in male adolescents with ODD (*p*<.001)

ABEP: Attentional Bias Execution Procedure; AESI: Academic Expectations Stress Inventory; AQ: Aggression Questionnaire; ATI: Ahvaz Test Anxiety Inventory; BPAQ: Buss and Perry Aggression Questionnaire; CBM: Cognitive Bias Modification; CDQ: Coping Disobedience Questionnaire; CG: Control Group; EAQ: Eating Attitude Questionnaire; ET: Experimental Trial; EMS: Early Maladaptive Schemas; GCST: Group Counseling Based on Schema Therapy; GEPT-ST: Good Enough Parenting Training with Schema Therapy; NG: Not Given; ODD: Oppositional Defiant Disorder; OS: Overeating Scale; PBMT: Parental Behavior Management Training; PD: Personality Disorder; PDT: Probe Dot Task; SAIAS-AB: Schema Assessment Inventory Through Activating Scenarios for Antisocial Behavior; SAQ: Pike and Wiseman Social Adjustment Questionnaire; SASA: Social Anxiety Scale for Adolescents; SCI: Schema Coping Inventory; SCQ: Social Competence Questionnaire; SDQ: Strengths and Difficulties Questionnaire; SDQ-PV: Strengths and Difficulties Questionnaire-Parent Version; SIC: Schema Inventory for Children; SMI: Schema Mode Inventory; SRE: Schema-Related Emotions; SRS: Self-Regulation Scale; SST: Social-Emotional Skills Training; ST: Schema Therapy; STCRS: Schema Therapy Competency Rating Scale; TG: Treatment Group; TSMI: Therapy Session Mode Inventory; YSQ-A: Young Schema Questionnaire for Adolescents; YSQ-SF3: Young Schema Questionnaire-Short Form 3; YSQSF: Young Schema Questionnaire-Short Form

### Parent-Based Schema Therapy Approaches

A total of eight studies examined parent-based schema therapy interventions, with one of these overlapping with the group-based schema therapy interventions mentioned above (Laious et al., 2024). The combined sample across these studies consisted of 502 parents or caregivers. The average number of sessions administered across the included studies was 11.3. Four out of eight studies reported improvements in child emotional and behavioral outcomes, with evidence suggesting that schema therapy supported parents in recognizing and modifying their own maladaptive schemas, thereby indirectly influencing child outcomes. (Beigi Harchegani & Ghazanfari, 2024; Solan et al., 2021; Jamshidi et al., 2023; Mawdsley et al., 2025).

Programs such as Good Enough Parenting (GEP) and Positive Parenting Program Training (PPPT) stood out for their emphasis on strengthening the parent–child relationship and promoting secure attachment patterns (Jamshidi et al., 2023; Louis et al., 2021). In a comparative study, both schema-based and compassion-based parenting trainings were found to be effective, demonstrating comparable levels of improvement (Qashqai et al., 2024), whereas another study found that both schema therapy and intensive short-term dynamic psychotherapy (ISTDP) reduced separation anxiety symptoms in children, though ISTDP showed relatively stronger effects (Beigi Harchegani & Ghazanfari, 2024).

Furthermore, Jamshidi et al. (2022) reported a significant decrease in parent–child conflict, with an effect size of η²*p* = .364 (Jamshidi et al., 2023). Collectively, these findings suggest that schema therapy is not only effective as an individual intervention for children and adolescents but also shows promising potential when applied within family-based and systemic frameworks, targeting parental schemas and relational dynamics to promote child well-being (Table 3).

**Table 3:** Parent-based schema therapy interventions for children and adolescents

Reference	Aim	Study design	Sample characteristics	Schema therapy format	Follow-up	Assessment tools	Results
Beigi Harchegani & Ghazanfari, (2024)	ISTDP & ST effectiveness for mothers of children (5–6 y) with separation anxiety	Quasi- ET	*n* = 45 mothers of children aged 5–6; Children: M(TG): 5.7 ± 1.4 M(CG): 5.2 ± 1.2 Mothers: M(TG): 38.6 ± 11.2, M(CG): 37.9 ± 10.8	Parent Coaching 10 weekly sessions (90 min each)	3 months	SCAS-P	Separation anxiety decreased in both treatments (*p*=.001); ISTDP more effective than schema therapy (*p*=.001, η²=0.90)
Solan et al., (2021)	ST strategies in parent program efficacy for preventing symptom relapse in ADHD children	Quasi- ET	*n* = 77; TG = 54, CG = 23; Female Ratio: 30%	Parent Coaching 25 weekly sessions (60 min each)	-	CBCL, DSM-5, Level 1–2 CC Symptom Measure for Attention Problems, HSQ, SSQ, TRF	Lower risk of symptom relapse in intervention group (*p*=.0004, d=0.43)
Van Wijk-Herbrink et al., (2021)	SafePath (ST-based milieu) effects in secure residential units vs. controls (1st year)	Quasi- ET	*n* = 87, 50 youth care worker, 139 adolescents	Schema Therapy based milieu-The SafePath intervention	-	LGWCI, GCI	Improvements in group climate (*p*=.001, η²=0.097), team functioning (*p*<.005, η²=0.125–0.13), and schema mode use (*p*<.001, η²=0.048) in SafePath units vs. control; no difference in aggression (*p*=.706)
Laious et al., (2024)	ST-based parent–child group program efficacy for preventing child psychopathology	Quasi- ET	*n* = 90; Female ratio: 47.8%; Age Range: 9–13; Mean Age: 11.2 ± 1.0	GST Children: 16 weekly sessions; Parents 10 biweekly sessions (90 min each)	-	SDQ-PV, SDQ, YSQ-SF3, SIC	Child EMS mostly decreased (*p*<.01, *r*=.19–0.27); many parent EMS decreased (*p*<.05, *r*=.16–0.25); reductions in child emotional symptoms (*p*=.001, *r*=.21), peer problems (*p*=.006, *r*=.22), total difficulties (*p*=.005, *r*=.22); prosocial behavior increased (*p*=.02, *r*=.19)
Jamshidi et al., (2023)	GEP & PPPT effectiveness on parent–child conflict in mothers of children with CD	Quasi- ET	*n* = 60; PPPT = 20, GEP = 20, CG = 20; Mothers’ Age Range: 25–45; Mean Age PPPT: 38.05 ± 9.08 Mean age GEP: 39.20 ± 8.38 Mean age CG: 36.65 ± 7.17	GEP 8 biweekly sessions (90 min each)	-	SCBSQ, PCCQ	GEP approach and PPPT reduced parent–child conflict (*p*=.001, η²*p*=.364)
Mawdsley et al., (2025)	CAREFREE (ST-based online group) feasibility & efficacy for carers of adults with severe or treatment resistant AN	Case Series	*n* = 53; Female Ratio:71.4%	Parent Coaching 12 weekly online sessions (75 min each)	3 months	EDSIS, FQ, SCORE-15, ECR-R, DASS-10, YPI-R2, SRS	Carer distress (*p*<.001, ICC=0.73), expressed emotion (*p*<.05, ICC=0.75–0.83), family functioning (*p*<.01), mood symptoms (*p*<.01, ICC=0.67–0.71), and attachment-related avoidance (*p*<.05, ICC=0.64) improved post-group and remained improved at follow-up
Louis et al., (2021)	User experiences of ST-based early parenting program for children’s core emotional needs & psychopathology prevention	Retrospective Exploratory Qualitative Study	*n* = 55 parents M: 46.55	Parent Coaching(GEP) 6 sessions (at least 21 h, or 3 full days)	-	Critical incident technique guided by semi-structured interview schedule	Participants reported high satisfaction; GEP improved parenting practices
Qashqai et al., (2024)	Schema- vs. compassion-based parenting training effectiveness on self-efficacy, self-concept & acceptance in mothers of children with internalizing problems	Quasi- ET	*n* = 45 (mothers of children aged 8 to 12 years)	Parent Coaching 8 sessions of 90 min each,	1 month	CBCL; CSC; PARQ-M; PSEQ	Schema-based and compassion-based parenting training improved parental self-efficacy, self-concept, and acceptance (*p*<.05); no difference between methods (Wilks’ Λ = 0.937, *p*<.001, η²≈0.06)

ADHD: Attention-Deficit/Hyperactivity Disorder; AN: Anorexia Nervosa; CBCL: Child Behavior Checklist; CC: Cross-Cutting; CD: Conduct Disorder CSC: Children’s Self-Concept Scale; DASS-10: Depression, Anxiety, and Stress Scale – Short Form; DSM-5: Diagnostic and Statistical Manual of Mental Disorders, Fifth Edition; ECR-R: Experiences in Close Relationships – Revised; EDSIS: Eating Disorder Symptom Impact Scale; EMS: Early Maladaptive Schemas; FQ: Family Questionnaire; GCI: Group Climate Inventory; GEP: Good Enough Parenting Programme; HSQ: Home Situation Questionnaire; ISTDP: Intensive Short-Term Dynamic Psychotherapy; LGWCI: Living Group Work Climate Inventory; MS-AN: Multi-Symptomatic Anorexia Nervosa; PARQ-M: Rohner’s Parental Rejection–Acceptance Questionnaire (Mother’s Form); PCCQ: Parent Child Conflict Questionnaire; PPPT: Positive Parenting Program Training; PSEQ: Dumka’s Parental Self-Efficacy Questionnaire; SCAS-P: Spence Children’s Anxiety Scale – Parent Form; SCORE-15: Systemic Clinical Outcome and Routine Evaluation – 15; SCBSQ: Sprafkin Children’s Behavioral Syndrome Questionnaire; SPBT: Schema-Enhanced Parent Behavior Therapy; SRS: Session Rating Scale; SSQ: School Situation Questionnaire; TRF: Teacher Report Form; YPI-R2: Young Parenting Inventory – Revised, Second Version

### Risk of Bias

According to the JBI Critical Appraisal Tool, most quasi-experimental studies demonstrated low to moderate methodological quality, with scores typically ranging between 6 and 9, reflecting certain limitations in study design and reporting. Only a small proportion of studies achieved ratings consistent with low risk of bias.

Notably, no RCTs met the revised inclusion criteria. One RCT (Bang & Lee, 2024) was identified at full-text screening but was excluded because the sample extended beyond the eligible youth age range and did not provide separable outcomes for children/adolescents. All included studies were quasi-experimental, case series, or other non-randomized designs. Consequently, the current evidence base should be considered preliminary and limited in its capacity to support strong causal inferences regarding the effectiveness of schema therapy in children and adolescents.

Overall, the methodological quality of the included studies can be characterized as moderate. While quasi-experimental designs provide valuable initial evidence, the absence of RCTs underscores the need for more rigorously designed studies to strengthen the evidentiary foundation of schema therapy in youth populations.

**Fig. 2:**
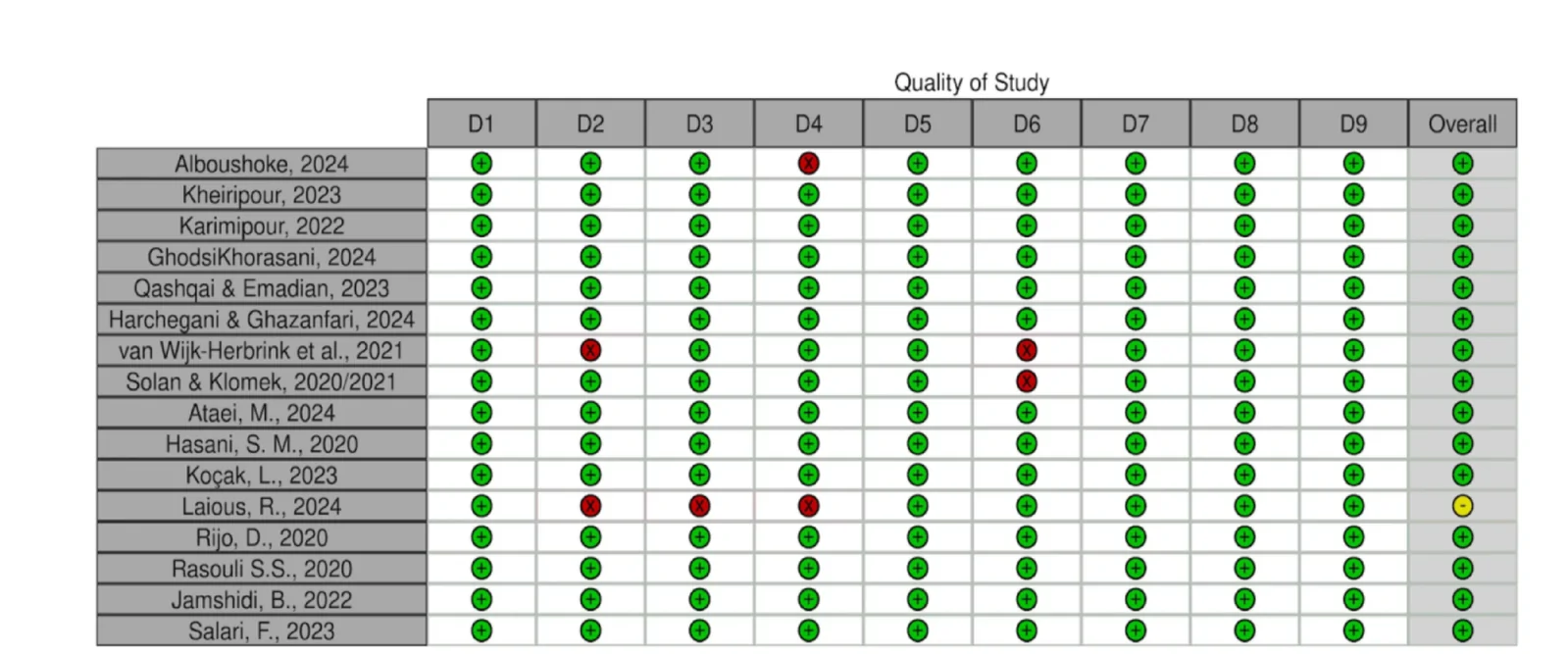
JBI critical appraisal checklist for quasi-experimental studies

**Fig. 3:**
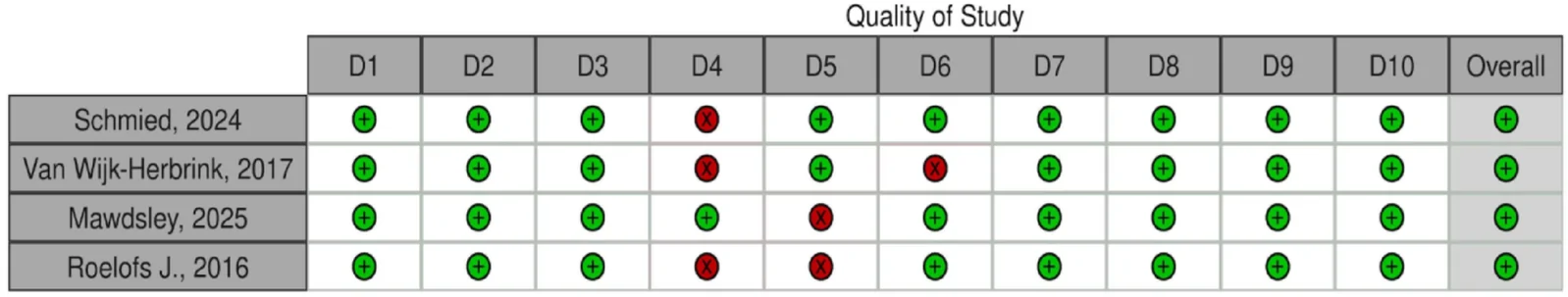
JBI critical appraisal checklist for case series

**Fig. 4:**
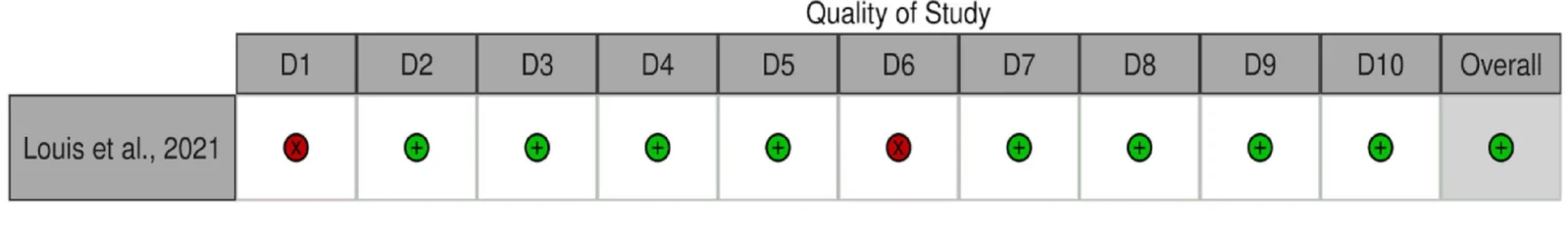
JBI critical appraisal checklist for qualitative study

The detailed results of the quality assessment are presented in Figs. 2, 3 and 4.

## Discussion

This systematic review synthesized current empirical findings on the effectiveness of schema therapy in children and adolescents across individual, group-based, and parent-focused formats. Overall, the included studies suggest that schema therapy offers promising outcomes in reducing emotional and behavioral difficulties, as well as maladaptive schemas, among young populations. While schema therapy is increasingly recognized for its utility across a range of psychological difficulties, it has been particularly emphasized in the treatment of depression, anxiety disorders, personality disorders, trauma-related conditions, and behavioral disturbances (Loose & Pietrowsky, 2016).

Although schema therapy can be applied transdiagnostically, the literature has traditionally focused more extensively on these aforementioned clinical groups. However, as observed in the present review, the scope of schema therapy in youth populations is expanding. The included studies span a wide range of issues beyond depression and anxiety—including internet addiction, eating disorders, social anxiety, oppositional defiant disorder (ODD) and personality features (Karimipour et al., 2022; Kheiripour et al., 2023; Koçak & Çelik, 2023; Rasouli Saravi et al., 2020). This pattern highlights the flexibility of schema therapy as a developmentally sensitive intervention that targets underlying emotional and cognitive patterns rather than disorder-specific symptom clusters. As such, its transdiagnostic applicability may be particularly advantageous in the treatment of children and adolescents, whose presentations often cut across conventional diagnostic categories.

Secondly, a closer examination of the studies included in this review reveals potential differences between schema therapy protocols applied to adults and those adapted for children and adolescents. Existing systematic reviews and clinical guidelines suggest that schema therapy—both for adults and younger populations—typically involves a higher number of sessions compared to other therapeutic approaches, often spanning a wide range of treatment durations (Bakos et al., 2015; Masley et al., 2012). This reflects the intensive and long-term nature of schema therapy, which is frequently characterized in the literature as a treatment modality that may extend over several months or even years (Bakos et al., 2015). However, studies focusing on children and adolescents suggest that shorter treatment durations can still yield significant therapeutic benefits, with many demonstrating sustained improvements at follow-up assessments (Kheiripour et al., 2023; Rasouli Saravi et al., 2020; Schmied et al., 2024). Several factors may account for this discrepancy. First, in childhood and early adolescence, early maladaptive schemas may still be in the process of formation; thus, they may be more malleable and less cognitively entrenched than in adulthood, allowing for effective intervention with fewer sessions. Second, early intervention through schema-based parent coaching may facilitate attitudinal changes in caregivers before maladaptive patterns become behaviorally consolidated, thereby enhancing therapeutic outcomes at an earlier developmental stage (Mawdsley et al., 2025). Third, schema therapy has been particularly emphasized as an effective treatment for personality disorders, which typically require more prolonged therapeutic engagement. However, personality disorder diagnoses are considered developmentally less stable and less valid in children and adolescents due to ongoing personality formation, identity development, and fluctuating socio-emotional patterns (Sharp, 2020). Consequently, ST-CA may more often target emotional and behavioral regulation rather than deeply ingrained personality structures, which could help explain the effectiveness of shorter interventions in younger populations (Bamelis et al., 2014).

Another noteworthy aspect emerging from the reviewed studies is the pattern of change in EMSs. Although the majority of studies in this population did not conduct detailed assessments using schema-specific measures, several did report improvements in EMS-related functioning (Laious et al., 2024; Roelofs et al., 2016). Interestingly, in some studies—such as Rijo (2020)—no significant changes were observed in EMSs, whereas significant improvements were reported in schema-related emotional experiences (Rijo et al., 2020). This suggests that emotional awareness and regulation skills may respond more quickly to schema-focused interventions, whereas the cognitive structures underpinning maladaptive schemas might require a longer period to reorganize and shift meaningfully. One might initially interpret the absence of EMS change in such cases as a result of limited treatment duration. However, in the study by Rijo (2020), the number of sessions was among the highest across the included studies, which challenges the assumption that session length alone explains the lack of cognitive schema modification (Rijo et al., 2020). Instead, this finding may reflect the hierarchical nature of change in schema therapy, where emotional restructuring precedes more enduring cognitive transformation (Young et al., 2006). It also raises important questions about measurement sensitivity and the temporal dynamics of change processes in schema therapy for adolescents, particularly those with entrenched personality features.

An additional compelling finding concerns the outcomes of parent–child schema-based interventions, in which improvements were reported not only in children’s emotional and behavioral functioning but also in parents’ early maladaptive schema scores (Laious et al., 2024). This suggests that schema therapy may facilitate restructuring not only at the individual level but also within broader family systems. From a systemic and developmental perspective, modifying maladaptive schemas in caregivers may indirectly improve children’s psychological outcomes by altering relational dynamics, emotional availability, and modeling behaviors (Mawdsley et al., 2025; Solan et al., 2021). Given that children’s schemas are often shaped within early attachment relationships, targeting parental schemas can be seen as an upstream intervention strategy with downstream effects on child well-being. These findings highlight the potential of schema therapy to operate through intergenerational pathways and underscore the importance of including family-level components in interventions for youth populations.

From a methodological standpoint, substantial heterogeneity was observed across the included studies. Compared to schema therapy trials in adult populations—which tend to rely on more standardized protocols and widely accepted outcome measures—child and adolescent studies frequently employed diverse, problem-specific instruments that were selected based on the presenting issue rather than a consistent measurement framework (Kheiripour et al., 2023; Salari et al., 2023; Schmied et al., 2024; Zhang et al., 2023). This lack of uniformity in assessment tools posed significant challenges for synthesizing findings and conducting meta-analytic procedures, ultimately limiting the ability to draw unified conclusions across studies. Additionally, the inclusion of small-sample studies reflects the developmental stage of schema therapy research in youth populations, where early feasibility and pilot trials remain common. This context should be considered when interpreting the strength of the current evidence base.

Furthermore, although age-appropriate schema measures are available and validated for use in youth populations, only a small proportion of the studies (approximately 17.4%) utilized such instruments to assess changes in EMSs. This underutilization hinders our understanding of the extent to which schema therapy targets its core mechanisms of change in younger populations and underscores the need for greater methodological rigor and standardization in future research. Employing consistent schema-specific measures across studies would not only improve comparability but also enhance theoretical clarity regarding treatment processes and outcomes.

While the recent review by Joshua et al. (2025) offers valuable preliminary insights into schema therapy for young people, the present review provides a broader and developmentally more targeted synthesis (Joshua et al., 2025). Unlike their review, which combines adolescents and young adults (12–30 years) into a single category, our work focuses specifically on children and adolescents, enabling clearer interpretation of schema therapy effects during key developmental periods. Furthermore, whereas Joshua et al. identified only 12 eligible studies, our review incorporates a larger and more diverse evidence base, including child-adapted protocols, school-based interventions, and family- and parent-focused schema therapy approaches—an essential consideration given the pivotal role of parenting and relational dynamics in youth mental health. By synthesizing child-adapted interventions across individual, group, and parent-based modalities, this review provides a developmentally refined framework that distinguishes youth-specific mechanisms of change and highlights the unique malleability of early schemas. This level of developmental specificity has not been highlighted in previous reviews and offers a clearer guide for both clinical decision-making and future intervention development. Finally, our methodological approach, grounded in PRISMA 2020 standards and systematic risk-of-bias assessment, offers enhanced rigor and clarity. Collectively, these features position our review as a more comprehensive and developmentally sensitive contribution to guiding schema therapy practice in child and adolescent populations.

Given the increasing demand for flexible and scalable youth mental health interventions, schema therapy may offer a clinically feasible option for diverse service settings. Shorter protocols, such as brief IR—an experiential technique involving guided reprocessing of distressing autobiographical memories—or focused mode work, which targets specific maladaptive emotional and coping states (e.g., vulnerable or avoidant modes), demonstrated meaningful improvements. These approaches aim to modify the emotional meaning of past experiences and promote more adaptive self-regulation within a limited number of sessions. Parent-focused schema interventions also align well with family-centered service structures and can be integrated into psychoeducation programs or community parenting workshops. Therapist training in schema therapy—particularly in experiential techniques—remains a key implementation factor and warrants further investigation in real-world settings.

In sum, this review highlights both the clinical promise and the ongoing challenges of applying schema therapy in child and adolescent populations. The evidence suggests that schema therapy, when developmentally adapted, appears promising in addressing a wide spectrum of emotional, behavioral, and relational difficulties through mechanisms. However, to fully harness its potential, future studies must aim for greater methodological standardization, clearer operationalization of schema constructs, and more longitudinal designs that capture the trajectory of change. Notably, despite the growing interest in schema therapy for youth, no RCTs met the revised inclusion criteria. One RCT (Bang & Lee, 2024) was identified at full-text screening but was excluded because the sample extended beyond the eligible youth age range and did not provide separable outcomes for children/adolescents. This gap substantially limits the ability to draw robust causal conclusions regarding its efficacy across diverse settings and populations. In the absence of randomized evidence, interpretations rely primarily on quasi-experimental and case series designs, which, although informative, provide a lower level of evidentiary strength. Accordingly, there remains a clear need for well-designed RCTs to establish the efficacy and generalizability of schema therapy in child and adolescent populations. Additionally, integrating parent-focused components and tailoring interventions to developmental needs may enhance both effectiveness and sustainability. As schema therapy continues to expand its reach in youth mental health, rigorous research efforts are essential to establish it as a developmentally sensitive, evidence-based modality with transdiagnostic relevance.

## Limitations

This review has several limitations that must be acknowledged. First, the overall number of available studies on schema therapy in children and adolescents remains relatively limited, and the included studies were all quasi-experimental or case series in design, with no RCTs meeting the revised inclusion criteria. This restricts the generalizability of the findings and weakens the ability to establish causal relationships. Although PsycINFO was not included in the search strategy, the databases selected (PubMed, Cochrane Library, Scopus, Web of Science, and Ovid MEDLINE) provide extensive and overlapping coverage of clinical psychology, psychiatry, and behavioral science journals. Given this substantial indexing overlap, the likelihood of systematically missing core schema therapy studies is considered low. Nonetheless, future updates may incorporate PsycINFO to further strengthen comprehensiveness. Second, the included studies demonstrated considerable methodological heterogeneity in terms of intervention duration, sample characteristics, outcome measures, and therapy formats. Such variability limited the capacity to conduct a full meta-analysis and precluded direct comparisons across subgroups. Furthermore, the inclusion of small case series may limit statistical robustness; therefore, conclusions regarding effectiveness should be viewed as provisional pending larger randomized trials. Third, many studies did not utilize validated schema-specific measures, making it difficult to assess whether observed improvements were driven by changes in the core constructs of schema therapy. Finally, the geographical distribution of studies was limited, with a substantial proportion conducted in Middle Eastern and Asian cultural contexts. Cultural norms related to parenting, emotional expression, and relational patterns may influence both schema development and treatment response, thereby limiting the broader cross-cultural generalizability of the findings. In addition, several studies lacked long-term follow-up data, restricting conclusions regarding the durability of treatment effects.

## Future Directions

To strengthen the empirical foundation of schema therapy in youth populations, future research should prioritize the implementation of methodologically rigorous RCTs. These studies should utilize standardized treatment protocols and validated outcome measures—including schema-specific instruments—to enable comparability across interventions and better isolate mechanisms of change. Longitudinal research designs are also needed to evaluate the sustainability of treatment effects and capture developmental trajectories over time. Future work should explore differential effects across age groups, diagnoses, and comorbidities to inform more personalized treatment planning. It is also important to investigate the role of therapist training and fidelity to schema therapy techniques, which may influence treatment outcomes. Moreover, the integration of parent- and family-based components should be systematically studied to determine their added value in enhancing therapeutic gains and supporting system-level change. Cross-cultural comparisons and develop culturally sensitive schema measures to clarify how cultural norms shape treatment processes. Such efforts will improve the broader applicability and ecological validity of schema therapy across diverse youth populations. Lastly, research on cost-effectiveness, therapist competency frameworks, and digital or blended schema therapy models may further enhance accessibility and sustainability in routine clinical services.

## Conclusion

In conclusion, this systematic review highlights the emerging potential of schema therapy as a developmentally sensitive, transdiagnostic intervention for children and adolescents. Despite the limited number of high-quality studies—particularly the absence of RCTs—the current evidence suggests that schema therapy may produce meaningful improvements in emotional, behavioral, and relational functioning across diverse clinical presentations. The flexibility of schema therapy in individual, group, and parent-based formats enhances its adaptability across various therapeutic contexts. Importantly, the effectiveness of shorter interventions in youth populations, as compared to more intensive protocols in adults, highlights the developmental malleability of early maladaptive schemas and suggests a need for age-tailored treatment planning. However, to solidify schema therapy’s position as an evidence-based approach in youth mental health, future research must address existing methodological inconsistencies, adopt more rigorous designs, and systematically assess schema-specific outcomes. With continued empirical support, schema therapy holds strong potential to become a cornerstone of integrative, mechanism-focused treatment for young populations.

## Data Availability

This study is a systematic review of previously published research. All data used in this study were extracted from the published articles included in the review, which are publicly available. The extracted datasets generated during the review are available from the corresponding author upon reasonable request.
